# Bacterial recognition pathways that lead to inflammasome activation

**DOI:** 10.1111/imr.12289

**Published:** 2015-04-16

**Authors:** Kelly M Storek, Denise M Monack

**Affiliations:** 1 Department of Microbiology and Immunology, Stanford UniversityStanford, CA, USA

**Keywords:** monocytes/macrophages, bacterial disease, inflammation, cytotoxicity, Toll-like receptors/pattern recognition receptors, signaling proteins

## Abstract

Inflammasomes are multi-protein signaling platforms that upon activation trigger the maturation of the pro-inflammatory cytokines, interleukin-1β (IL-1β) and IL-18, and cell death. Inflammasome sensors detect microbial and host-derived molecules. Here, we review the mechanisms of inflammasome activation triggered by bacterial infection, primarily focusing on two model intracellular bacterial pathogens, *Francisella novicida* and *Salmonella typhimurium*. We discuss the complex relationship between bacterial recognition through direct and indirect detection by inflammasome sensors. We highlight regulation mechanisms that potentiate or limit inflammasome activation. We discuss the importance of caspase-1 and caspase-11 in host defense, and we examine the downstream consequences of inflammasome activation within the context of bacterial infections.

## Introduction

The innate immune system has evolved numerous defense mechanisms to quickly recognize and respond to infection (1). A critical response for an effective host response to infection is the induction of inflammation. While inflammation is a critical response to infections, this process must be carefully regulated to prevent excessive tissue injury. Dysregulation can result in a multitude of immune disorders including atherosclerosis and inflammatory bowel disease (IBD) (2). Recognition and coordination of signals are important for infection resolution, whereas aberrant regulation can lead to disease.

The initiation of inflammation begins when host pattern recognition receptors (PRRs) detect conserved molecular signatures associated with the presence of a pathogen, called pathogen-associated molecular patterns (PAMPs), or host-derived molecules that signal the presence of tissue injury, called damage-associated molecular patterns (DAMPs). To date, five classes of PRRs have been described. Toll-like receptors (TLRs) and C-type lectin receptors recognize PAMPs and DAMPs located extracellularly or within vacuolar compartments, while retinoic acid-inducible gene (RIG)-I-like receptors (RLRs), NOD-like receptors (NLRs), and AIM2-like receptors (ALRs) recognize PAMPs and DAMPs located within the cytoplasm. Upon sensing PAMPs or DAMPs, PRRs elicit a host defense response through several conserved signaling pathways. Some PRRs promote a pro-inflammatory and antimicrobial transcriptional response through the nuclear factor κB (NFκB) or interferon-regulatory factors (IRFs) pathways. Other PRRs promote host defense through the assembly of cytoplasmic signaling complexes, coined in 2002 by Martinon *et al*. as ‘inflammasomes’ (3).

Inflammasomes are macromolecular complexes that assemble in the cytoplasm to ultimately activate caspase-1. They typically consist of an NLR or ALR sensor that recognizes a ligand, an adapter protein, ASC, and the cysteine protease caspase-1, which is a key mediator in innate immune defense (4). The NLR family encodes 23 members in humans and at least 34 members in mice, and the ALR family encodes 4 members in humans and 13 members in mice. Upon ligand recognition, the sensor associates with ASC, which recruits caspase-1. Activation of caspase-1 leads to the proteolytic cleavage and secretion of the pro-inflammatory cytokines, interleukin-1β (IL-1β) and IL-18, as well as the release of leaderless proteins involved in tissue repair and cytoprotection (5,6). In addition, caspase-1 activation results in a rapid, pro-inflammatory form of cell death called pyroptosis (7). Consequently, inflammasome assembly is tightly regulated to prevent aberrant pro-inflammatory response and cell death.

In this review, we highlight new insights on inflammasome regulation, composition, and ligand recognition, as well as the downstream consequences of inflammasome activation, primarily in the context of bacterial infections.

## Bacterial ligands and corresponding inflammasome host receptors

**Fig. 1:**
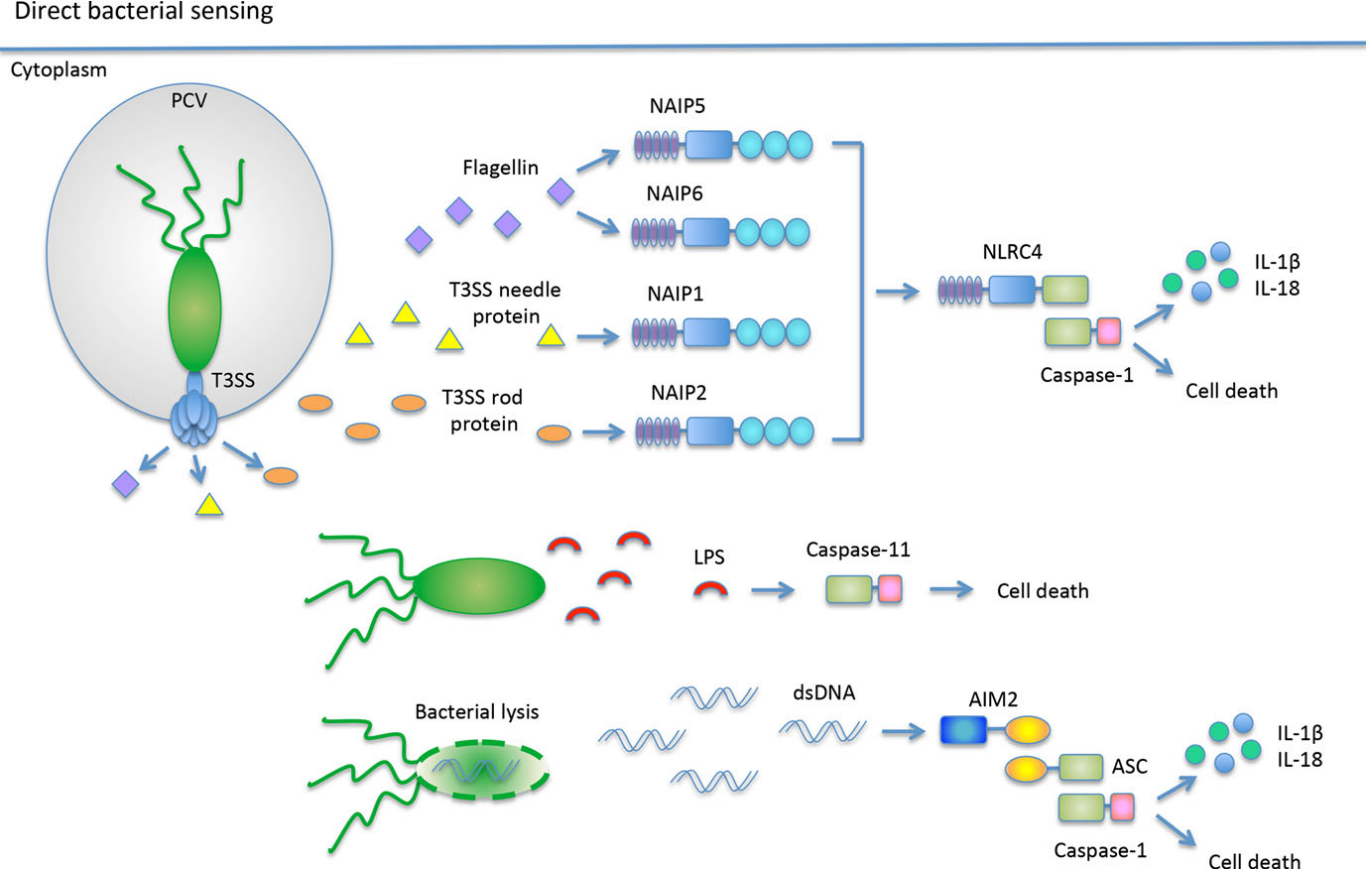
Direct bacterial sensing by inflammasomes A subset of inflammasome receptors directly recognizes bacterial components. The receptors, NAIP5 and NAIP6, recognize bacterial flagellin, while NAIP1 and NAIP2 recognize the T3SS needle protein and rod protein, respectively. Upon ligand recognition, the NAIPs interact with NLRC4 to activate caspase-1 resulting in cell death and cytokine processing and secretion. AIM2 functions as a DNA sensor, detecting DNA from lysed cytosolic bacteria such as *F. novicida*. AIM2 interacts with ASC to activate caspase-1. Bacterial LPS is directly sensed by caspse-11 to elicit cell death.

**Fig. 2:**
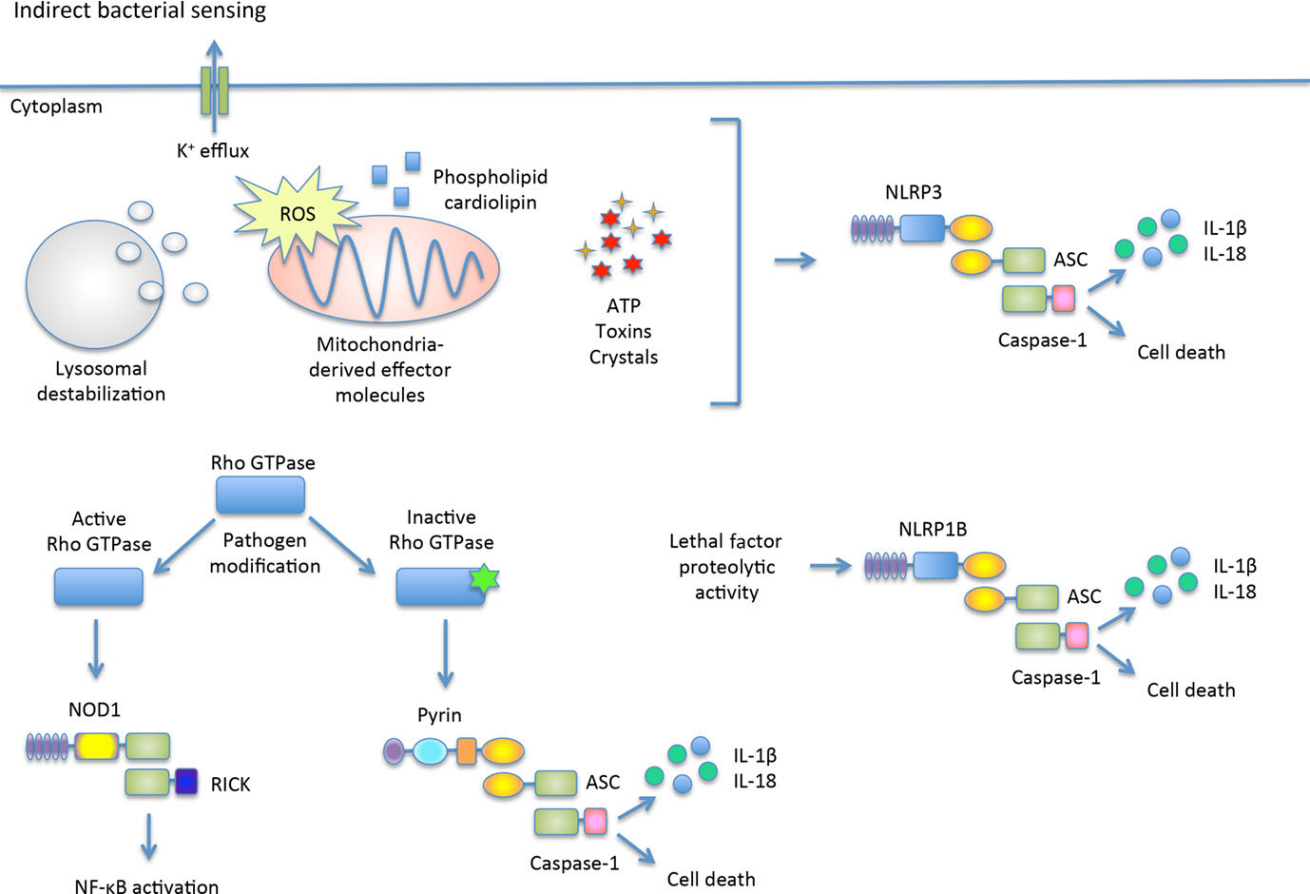
Indirect bacterial sensing by inflammasomes A subset of inflammasome receptors indirectly recognizes bacterial infection by responding to host alterations typically found during an infection. These are referred to as ‘Patterns of Pathogenicity’. NLRP3 responds to a variety of stimuli including host cell changes that enhance potassium efflux, elevate reactive oxygen species (ROS) and mediate lysosomal destabilization. NLRP1B detects the proteolytic activity of *Bacillus anthracis* lethal factor (LF) protease. Pyrin detects the inactivation of RHOA/B/C GTPases, while NOD1 detects activated Rho GTPases and induces a NF-κB-dependent inflammatory response. NLRP3, Pyrin, and NLRP1B each interact with ASC to activate caspase-1.

A variety of bacterial infections, including *Salmonella typhimurium*, *Francisella* spp., *Burkholderia* spp., *Shigella flexneri*, and *Escherichia coli*, can elicit robust inflammasome activation in human monocyte-derived and mouse bone marrow-derived macrophages (BMDMs) (8). The mechanisms by which infections with these pathogens lead to inflammasome activation have been under intensive investigation. Bacterial pathogens have been used to help characterize the factors involved in inflammasome assembly, composition and activation. A major advantage to using bacterial models is that both bacterial and eukaryotic genetics can be harnessed to unravel the molecular mechanisms involved in inflammasome activities. How inflammasomes sense bacterial infections can be divided into two categories: (i) direct microbial sensing (*Fig.* 1) and (ii) indirect microbial sensing (*Fig.* 2).

### Flagellin

Bacterial flagella are long, complex organelles that protrude well beyond the bacterial cell wall, reaching up to 10 μm in length. While well known for their ability to generate bacterial movement via swimming and swarming motilities, flagella are also important in bacterial surface adhesion, biofilm formation, and pathogenesis (9). Depending on the bacterial species, the number of flagellum and the location of the flagellum can differ (10). For example, *Pseudomonas aeruginosa* produces a single, polar flagellum, whereas *Salmonella enterica* serovar Typhimurium produces six to eight peritrichous flagellum (11,12). In addition, some bacterial species (such as *Vibrio parahaemolyticus*) have the genetic capacity to produce two unique flagella (12). Over 50 genes divided into multiple operons are required for the assembly and function of the bacterial flagellum, including genes encoding structural subunits, regulatory factors, motor force generators, chemotactic proteins, and export proteins (10).

The flagellum filament extending from the bacterial surface consists primarily of repeating structural subunits, known as flagellin. The flagellin monomer, but not the filament, activates TLR5 signaling and leads to NF-κB and IL-8 secretion (11). In addition, two laboratories identified a TLR5-independent pathway for flagellin detection using a *S. typhimurium* infection model (13,14). *S. typhimurium* mutants that either lack or have altered flagella do not stimulate caspase-1 or IL-1β secretion (13,14). Rather than acting through the TLR5 pathway, caspase-1 activation and IL-1β secretion induced by *S. typhimurium* requires the cytoplasmic sensor NLRC4 (also known as Ipaf), revealing two independent pathways for flagellin detection (13,14). To evade detection, *S. typhimurium* represses flagellin expression *in vivo*. *S. typhimurium* strains engineered to continuously express flagellin hyperactivated the NLRC4 inflammasome and are severely attenuated in a mouse model (15). Experiments using purified flagellin confirmed flagellin to be a stimulatory ligand for NLRC4 inflammasome activation, but no direct interaction was found (13,14). Rather, in mice, neuronal apoptosis inhibitory protein 5 (NAIP5) and NAIP6 are the direct sensors of flagellin and act as co-receptors for NLRC4 activation (16–18) (*Fig.* 1).

In humans, only one NAIP orthologue exists (hNAIP) and initial studies demonstrated that hNAIP does not bind purified flagellin (17). Some human cell lines (such as U937 monocyte-derived macrophages) do not induce inflammasome activation in response to cytosolic flagellin (17). These results are likely due to human cell lines expressing very low levels of hNAIP (19). In contrast, primary human cells, including monocyte-derived macrophages, monocyte-derived dendritic cells, and neutrophils, express hNAIP (19). Moreover, studies using primary human macrophages demonstrated that flagellin-deficient strains of *L. pneumophila* replicate more efficiently compared to wildtype bacteria and this enhanced replication is dependent on NLRC4 (20). Similarly, work from our laboratory demonstrated that primary human macrophages infected with flagellin-deficient strains of *S. typhimurium* elicited a reduced inflammasome response compared to the wildtype *S. typhimurium* (authors’ unpublished data). Determining how bacterial flagellin may activate the inflammasome in human cells is an area of intense research. Moreover, examining the role of flagella in primary human cells using bacterial strains that are specifically human-adapted, such as *S. typhi*, may reveal novel human-specific pathways involved in pathogen recognition.

### Type III secretion system (T3SS) rod and needle proteins

Many Gram-negative bacteria that interact with eukaryotic cells are equipped with a protein secretion apparatus known as the type III secretion system (T3SS) (21). The T3SS facilitates the delivery of bacterial effector proteins across the cell membrane into the host cytosol (21). For many pathogens, the T3SS is an essential virulence factor. Depending on the bacterial species, the T3SS activity can prompt invasion in non-phagocytic cells, downregulate pro-inflammatory responses, alter intracellular trafficking pathways, trigger apoptosis, and prevent autophagy (22).

The T3SS is a large proteinaceous structure that extends from the bacterial cell reaching up to 80 nm in length and 8 nm in width (23). The T3SS is thought to form a continuous channel for bacterial effector proteins to travel from the bacterial cytosol into the host cytosol. While the effector proteins secreted from the T3SS are highly diverse, the structural apparatus itself is relatively conserved (21). The T3SS apparatus is a hollow multiprotein complex composed of a basal body with an inner rod, which is a cylindrical substructure that connects the base to the needle protein. During assembly, the secretion system exports the rod subunits into the interior of the basal body, where they polymerize and extend through the length of the T3SS. Subsequently, needle proteins are exported to directly connect the bacterium to the host cell and facilitate effector transfer (24). In the case of *S. typhimurium*, the inner rod protein is PrgJ and the needle protein is PrgI (24).

Initial studies using *S. typhimurium* as a model system determined that the T3SS rod protein, PrgJ, encoded from *Salmonella* pathogenicity island-1 (SPI1) triggers an inflammasome response dependent on NLRC4 (25). These results were initially perplexing, because NLRC4 was also required for recognition of the structurally distinct flagellin protein (13,14). More recently, studies clarified this discrepancy and identified NAIP2 as a direct sensor of the conserved family of T3SS rod proteins (17). Upon sensing T3SS rod proteins, NAIP2 promotes physical association with NLRC4, resulting in NLRC4 inflammasome activation (*Fig.* 1) (17). Additionally, a second group demonstrated that murine NAIP1 recognizes the bacterial T3SS needle proteins, MxiH from *Shigella flexneri* and EprI from *E. coli* (26). Like NAIP2, ligand bound NAIP1 mediates the formation and activation of the NLRC4 inflammasome complex (26). Bone marrow-derived dendritic cells (BMDCs) are more reactive to recognizing the T3SS needle protein during a *S. flexneri* infection as compared with BMDMs (26). In a follow-up study, BMDMs were shown to express insufficiently low levels of NAIP1 in the resting state, suggesting cell-type differences in inflammasome regulation (27).

Although the sole human NAIP (hNAIP) is highly homologous (sharing 68% identity at the amino acid level) to murine NAIP5, initial studies using human U937 macrophages demonstrated that hNAIP does not respond to flagellin proteins (17). Instead, hNAIP appears to have homologous function to NAIP1 and specifically recognizes the T3SS needle proteins, CprI from *Chromobacterium violaceum*, MxiH from *Shigella flexneri*, and EprI from *E. coli*, but not TTSS rod proteins from *S. typhimurium* and *Burkholderia thailandensis* (17,26). CprI interaction with hNAIP stimulates NLRC4-dependent inflammasome activation in human U937 monocyte-derived macrophages (17). These experiments demonstrate that hNAIP recognizes bacterial needle proteins. One potential explanation as to why hNAIP does not recognize the TTSS rod protein is that recognizing only one component of the TTSS may be sufficient for inflammasome activation. In addition, the needle protein, which is essential for T3SS function, has direct access to the host cytosol and may be more prone to recognition. Future studies will be important to determine if and/or what other proteins are involved in recognizing bacterial flagellin in humans.

### dsDNA

While recognition of nucleic acids has been extensively described for viral infections, recent research has demonstrated that bacterial nucleic acids also robustly activate innate immune pathways, including the type I interferon response and inflammasome response (28). In the quest to identify novel inflammasome receptors through targeted screens, absent in melanoma 2 (AIM2) was identified as a cytosolic receptor for double stranded (dsDNA) and an inflammasome activator (29–32). AIM2 is a member of the PYHIN [Pyrin and homopoietic IFN-inducible nuclear proteins with a 200-amino acid motif (HIN-200)] protein family. Upon dsDNA engagement via the HIN-200 domain, the N-terminal pyrin domain associates with ASC through homotypic pyrin–pyrin interactions (33) (*Fig.* 1). AIM2 is the first example of a non-NLR family protein to initiate inflammasome activation.

The dsDNA-AIM2 inflammasome pathway is important for host cells to detect stealth bacterial invaders that lack highly stimulatory ligands, such as flagellin, T3SS, and Lipopolysaccharide (LPS). One such bacterium is *F. novicida*. Host cells infected with *F. novicida* elicits a robust caspase-1-dependent inflammasome response that was not dependent on NLRP3 or NLRC4 inflammasomes (34). The discovery of AIM2 as a novel inflammasome receptor led us to examine its role during infection with *F. novicida*. Caspase-1 cleavage, IL-1β secretion, and pyroptotic cell death were absent in *AIM2*^*−/−*^ BMDMs infected with *F. novicida* (30,35). Moreover, *AIM2*-deficient mice, like caspase-1-deficient mice, display increased susceptibility to *F. novicida* infection compared with wildtype mice (30,35). In addition to *F. novicida*, AIM2 is important for inflammasome activation during infection with *Listeria monocytogenes*, *Mycobacterium tuberculosis*, *Porphyromonas gingivalis*, *Streptococcus pneumoniae*, vaccinia virus, and mouse cytomegalovirus, but not *S. typhimurium* infection (35–39).

How bacterial dsDNA is being sensed in the host cell cytosol was initially perplexing. Unlike viral infections, it is presumed that dsDNA is safely enclosed within the bacterial membrane and inaccessible to host cell receptors during infection. In addition, the location of dsDNA is important, since only cytosolic dsDNA triggered AIM2 inflammasome activation (29). Interestingly, two bacterial pathogens that stimulate the AIM2 pathway, *F. novicida* and *L. monocytogenes,* replicate in the cytosol of host cells (40,41). Confocal microscopy revealed that a small subpopulation of wildtype *F. novicida* appear to lyse in the cytosol of macrophages, as dsDNA was no longer contained within the defined cell wall of the bacterium but detected near the lysed bacterium (35). In addition, only the dsDNA not contained within the bacterial cell wall co-localized with endogenous AIM2 and this AIM2:DNA complex is required for ASC recruitment (35). These results suggest that bacterial lysis can lead to AIM2 inflammasome activation. Similar to *F. novicida*, a subset of wildtype *L. monocytogenes* lyse in the macrophage cytosol, release dsDNA, and stimulate the AIM2 inflammasome (36,42–44).

The classic genetic experiment to create a bacterial mutant lacking the stimulatory ligand is not possible for the AIM2 inflammasome, since DNA is essential for bacterial viability. However, we and others (34,45–48) identified *F. novicida* mutants that induce higher host cell cytotoxicity by hyperstimulating the AIM2 inflammasome. Many of the identified hypercytotoxic *F. novicida* strains contain mutations in genes whose predicted products are outer membrane proteins (FTT0584, MviN, RipA, FopA, and FTN1217) or products involved in LPS biosynthesis (WbtA and LpxH) (48). On the basis of the types of mutations identified, we hypothesized that the hypercytotoxic mutants had reduced cell wall integrity and, as such, would be subject to enhanced intracellular lysis. In support of this hypothesis, these mutants exhibit aberrant morphologies when grown in minimal media (48). Additionally, the hypercytotoxic phenotype for all mutants is dependent on the presence of AIM2 and the ability of the mutants to access the cytosol (48). These results suggest that more cytosolic dsDNA is present and stimulating the AIM2 inflammasome. To further support this claim, the hypercytotoxic mutants release higher levels of bacterial DNA into the host cytosol compared to wildtype *F. novicida*, as measured by confocal microscopy and the delivery of a reporter plasmid (48,49). Recently, the clustered, regularly interspaced, short palindromic repeats-CRISPR associated (CRISPR-Cas) system in *F. novicida*, a prokaryotic defense system against foreign nucleic acids, was found to enhance the integrity of the bacterial envelope through the regulation of a bacterial lipoprotein (50). This CRISPR-Cas system is important for evading AIM2 inflammasome activation and host cell death, providing additional support for the hypothesis that bacterial membrane stability is important to avoid intracellular lysis and AIM2 inflammasome activation (50). These results have implications for other bacterial pathogens indicating that mutations in membrane-associated proteins or cell wall biosynthesis may affect the stability and/or integrity of the bacterial membrane and potentially increase the pathogen's susceptibility to lysis and subsequent release of PAMPs.

### LPS

LPS is a major outer membrane component ubiquitously expressed in gram-negative bacteria. LPS consists of several well-conserved domains including a hydrophobic domain known as lipid A (or endotoxin), a core oligosaccharide, and a repetitive glycan polymer called the O-antigen. The LPS layer is essential for bacterial viability and functions as a protective permeability barrier from external stress factors such as bile salts, lysozyme, and other antimicrobial agents (51). In addition, the O-antigen plays an important role in effective colonization of host tissues and resistance to complement-mediated killing (51).

LPS is a potent activator of the innate immune response and, at relatively low doses, can cause septic shock (52). Until recently, LPS was thought to exclusively signal through the TLR4 and myeloid differentiation factor 2 (MD2) cell-surface receptor complex to stimulate signaling pathways that lead to the production of pro-inflammatory cytokines and type I IFNs (53). However, there were some hints of TLR4-independent sensing of LPS. For example, several studies, including our own, linked cytosolic activation of the non-canonical caspase-11 inflammasome to infection by a broad range of gram-negative bacteria (*S. typhimurium*, *E. coli*, *Vibrio cholerae*, *Citrobacter rodentium*, and *Legionella pneumophila*), but not gram-positive bacteria (54–57). Caspase-11 inflammasome activation results in pyroptotic cell death, but unlike caspase-1 inflammasome activation, is not required for the secretion of the pro-inflammatory cytokines (IL-1β or IL-18) (55). Despite the association of Gram-negative bacterial infections to caspase-11 inflammasome activation, LPS alone was not sufficient for activation (58). Rather, LPS had to gain access to the cytosol before it could activate the caspase-11 inflammasome (59). While activation of caspase-11 by cytosolic LPS could be uncoupled from TLR4 signaling, Toll/IL-1 receptor domain-containing adapter inducing IFN-β (TRIF)-mediated type I IFN production was essential for caspase-11 activity (55,59). Further experiments determined the hexa-acyl lipid A moiety was the LPS component essential to activate caspase-11 (59). Interestingly, the hexa-acyl lipid A moiety is the same activator for TLR4.

There are a number of differences between caspase-1 inflammasome activation and caspase-11 inflammasome activation. One example is that caspase-11 activity does not depend on the adapter protein ASC (60). In addition, purified caspase-11 and the human homolog Caspase-4 can directly bind LPS and lipid A with high specificity and affinity (60). Upon LPS or lipid A binding, caspase-4 and caspase-11 oligomerize, which is necessary for catalytic activity (60). Mutational analysis determined that the CARD domain in caspase-11 mediates LPS recognition and oligomerization (60) (*Fig.* 1). Although this body of work indicates a new role for the inflammatory caspases, caspase-11 and Caspase-4, as direct PAMP sensors, whether this occurs in the context of a bacterial infection remains to be determined.

### Patterns of pathogenesis

The innate immune system is essential for the detection and elimination of bacteria. However, how the innate immune system differentiates between pathogenic and commensal bacteria is a perplexing question, as many stimulatory PAMPs (such as LPS, DNA, and flagellin) are conserved between both commensal and pathogenic bacteria. One notable difference is that pathogenic bacteria employ effector molecules that manipulate the host cell to promote a more favorable environment. A number of groups recently identified host factors that are important for recognizing ‘Patterns of Pathogenicity’ (*Fig.* 2). For example, the NLRP3 receptor detects ion fluxes elicited by the membrane-damaging pore-forming toxin, nigericin (61). In addition, NLRP1B senses the proteolytic activity of *Bacillus anthracis* lethal factor (LF) protease, a key secreted virulence factor (62). NLRP3 and NLRP1B are two examples in which host receptors do not directly sense the bacterium. Rather, these receptors are sensing host modifications that are a direct result of an infection by a bacterial pathogen. Recognition of these host modifications by NLRP3 and NLRP1B results in caspase-1 activity (62).

Two recent reports (63,64) described the sensing of an additional pattern of pathogenicity: the modification of host regulatory GTPases, specifically Rho-GTPases. Rho-GTPases are molecular switches essential for cytoskeleton regulation (65). The cytoskeleton aids in limiting pathogen invasion and dissemination in tissues by primarily functioning as a physical barrier, but the cytoskeleton is also important in the migration, phagocytosis, cell growth, and cell signaling of immune cells (65). Because of these functions, Rho-GTPases are a frequent target of effector proteins by bacterial pathogens (65). Both the activation and inactivation of Rho GTPases by bacterial pathogens have been described (65). Pathogens often activate Rho GTPases to gain access to the host cytosol (66,67). In contrast, pathogens that inactivate Rho GTPases through ADP-ribosylation, glucosylation, adenylylation, or proteolysis frequently disrupt the actin cytoskeleton and cause cell death (65). *S. typhimurium* effector protein SopE activates three Rho GTPases: RAC1, CDC42, and RHOA (63). Activation of these Rho GTPases by *S. typhimurium* triggers the NOD1 signaling pathway that induces a NF-κB-dependent inflammatory response (63). In contrast, a novel PRR, Pyrin, senses the inactivation of RHOA/B/C GTPases, but not other Ras GTPases (64). Pyrin was also demonstrated to mediate caspase-1 inflammasome activation (64). Since Pyrin responds to a variety of GTPase modifications including ADP-ribosylation, glucosylation, and adenylylation occurring on different residues, it was reasoned that Pyrin likely senses the downstream consequences of GTPase inactivation rather than a direct modification on the GTPase itself (64). These studies identify novel mechanisms that the host uses to sense the consequences of pathogen-derived toxins, enzymes, or proteases. Through these mechanisms, and presumably many more, the host is capable of distinguishing pathogens from commensals.

## Caspase-1 in bacterial infections

Caspase-1 is the best-characterized inflammatory caspase and is the central effector protein of the inflammasome. Upon activation, caspase-1 cleaves the inactive form of IL-18 and IL-1β to their mature bioactive forms. In addition, caspase-1 also directs an inflammatory form of cell death called pyroptosis. Many examples demonstrate a critical role for caspase-1 in the defense against a broad spectrum of bacterial pathogens.

*F. novicida* is a non-flagellated, Gram-negative bacterium that replicates robustly within the cytosol of host cells. *F. novicida* initially resides within a *Francisella* containing vacuole (FCV) upon phagocytosis into host cells but quickly escapes into the host cytosol through a mechanism that is not well understood. Vacuolar escape requires a type 6-like secretion system encoded on the *Francisella* pathogenicity island (FPI) (68). *FPI* mutants are considerably attenuated *in vivo*, indicating the importance of cytosolic access for full virulence (45). Once in the cytosol, *F. novicida* activates caspase-1 (34). Caspase-1-dependent response to *Francisella* is essential for host resistance *in vivo* as *Casp1*^*−/−*^ mice succumbed faster to infection and carried higher bacterial burdens in the spleen and liver (34). Caspase-1 activation is also important in resisting infection against *Shigella*, *Legionella*, *Mycobacterium*, and *Listeria* (69–72).

*S. typhimurium* is a model intracellular pathogen often used to characterize host-pathogen interactions. C57BL/6 *Casp1*^*−/−*^ mice are markedly more susceptible to oral colonization by wildtype *S. typhimurium* (55,73,74). In addition, significantly higher bacterial burdens were found in systemic organs including the spleen and mesenteric lymph nodes (73).

*In vitro* experiments of *S. typhimurium* infections of C57BL/6 BMDMs require caspase-1 and NLRC4 to elicit cell death and IL-1β secretion (75). Surprisingly, in contrast to C57BL/6 *Casp1*^*−/−*^ mice, C57BL/6 *Nlrc4*^*−/−*^ mice are phenotypically comparable to wildtype mice and are not hypersusceptible to *S. typhimurium* infection (73). Interestingly, BALB/c *Nlrc4*^*−/−*^ mice are more susceptible to *S. typhimurium* when infected orally (76). This discrepancy of NLRC4 contribution *in vivo* to *S. typhimurium* resistance is most likely due to large inherent differences in inflammatory responses between C57BL/6 and BALB/c backgrounds. However, the inconsistency between the *in vitro* and *in vivo* C57BL/6 data was later attributed to differences in the host response to two distinct T3SSs encoded on *Salmonella* pathogenicity islands: SPI-1 and SPI-2 (74). When *S. typhimurium* is grown in log-phase, SPI-1, and flagellin expression is up-regulated, which stimulates the NLRC4-caspase-1 inflammasome (75). However, during a mouse infection, *S. typhimurium* down regulates SPI-1 expression. We found that under conditions where SPI-1 is downregulated (*in vivo* infection and *in vitro* stationary growth phase), both NLRC4- and NLRP3-caspase-1-dependent pathways were activated (74,77). SPI-2 and flagellin were the necessary *Salmonella* factors required to activate the NLRC4 inflammasome (74). However, to date, the mechanism of NLRP3 activation during a *S. typhimurium* infection remains a mystery. *S. typhimurium* SPI-2 mutants still activate the NLRP3 inflammasome (74). This is interesting given previous work demonstrating that *SPI-2* mutants are defective for intracellular replication and thus, NLRP3 may be capable of responding to non-replicating bacterial populations (78). Future work characterizing the role of NLRP3 in recognizing bacterial persisters will be an exciting avenue to pursue.

## Caspase-11 in bacterial infections

In 2011, the importance of the fact that the widely distributed *Casp1*^*−/−*^ mouse also lacked functional caspase-11 was highlighted (58). The 129 embryonic stem cells used to generate the original *Casp1*^*−/−*^ mouse contain a naturally occurring 5 bp deletion that disrupts exon splicing (58). Unfortunately, due to the close proximity of caspase-1 and caspase-11, segregating the caspase-1 mutant from the inactive form of caspase-11 was highly unlikely. This realization prompted a reassessment of the independent roles of caspase-1 and caspase-11 in bacterial infections. Initial studies generated *Casp11*^*−/−*^ mice and demonstrated that *E. coli* infection of BMDMs activates caspase-11 (58). In addition, *Casp11*^*−/−*^ BMDMs did not die when infected with live *E. coli*, *C. rodentium*, and *V. cholerae*, but did die in response to *F. novicida* or *P. aeruginosa* infection (58). Finally, these studies demonstrated that LPS-induced sepsis was solely dependent on caspase-11 (58,79).

Early studies demonstrated that *S. typhimurium* infection activates caspase-1 in peritoneal macrophages and BMDMs (75,80). In addition, *Casp1*^*−/−*^ mice were found to be more susceptible to *S. typhimurium* infection, as measured by an increase in bacterial burden in the Peyer's patches, mesenteric lymp hnodes, spleen, and liver, as well as reduced survival (73,74). However, the functions of caspase-1 and caspase-11 during *S. typhimurium* infection was not known. With the availability of the single knockout mice (58), we set out to tease apart the function of caspase-1 and caspase-11 during *S. typhimurium* infection.

*S. typhimurium* activates the NLRC4-caspase-1 inflammasome either through the secretion of PrgJ, which is the T3SS rod protein encoded by *Salmonella* pathogenicity island 1 (SPI-1), or flagellin subunits by way of the SPI-1 or SPI-2 T3SS (13,25,74,75). Furthermore, we found that when *S. typhimurium* SPI-1 is downregulated (stationary phase growth), caspase-1 activity is dramatically delayed, occurring between 12 and 17 h postinfection (74). Under these conditions, both the NLRC4 and NLRP3 inflammasomes are activated (74). Taking advantage of mutant *S. typhimurium* strains revealed that flagellin secreted by SPI-2 activates NLRC4, whereas NLRP3 responds primarily to an unidentified T3SS-independent stimulus (74). To examine the role of caspase-11 during a *S. typhimurium* infection, we utilized culturing conditions that minimized caspase-1 activation by infecting with flagella-deficient *S. typhimurium* (Δ*fliAB*Δ*fliC*) grown to stationary phase (55). BMDMs infected with Δ*fliAB*Δ*fliC* led to the processing of caspase-11 (55). In addition, Δ*fliAB*Δ*fliC* -induced cell death in wildtype BMDMs is dependent on caspase-11 as both *Casp11*^*−/−*^ and *Casp1*^*−/−*^*Casp11*^*−/−*^ BMDMs have similarly reduced levels of cell death (55).

The contribution of caspase-11 to pro-inflammatory cytokine secretion is less clear. *S. typhimurium* infections of *Casp1*^*−/−*^*Casp11*^*−/−*^ BMDMs are completely defective for IL-1β secretion, but *Casp11*^*−/−*^ BMDMs still elicit an IL-1β response, albeit at approximately one-third the level of wildtype BMDMs (55). Defective IL-1β secretion is not due to lower expression of pro-IL-1β, as *Casp11*^*−/−*^ and wildtype BMDMs have similar expression when infected with *S. typhimurium* (55). These results parallel the previous observation that caspase-1 is essential for the processing and secretion of IL-1β, while caspase-11 potentiated caspase-1 activation downstream of the NLRP3 inflammasome (58).

We next explored the role of caspase-11 during an oral *S. typhimurium* infection *in vivo*. Similar to earlier reports, *Casp1*^*−/−*^*Casp11*^*−/−*^ mice have higher bacterial loads in the spleen, liver, and mesenteric lymph nodes (55,73). Surprisingly, *Casp11*^*−/−*^ and wildtype mice have similar bacterial loads. In contrast, the levels of bacteria in *Casp1*^*−/−*^ mice are significantly higher than the *Casp1*^*−/−*^*Casp11*^*−/−*^ mice (55). These results indicate that caspase-11 expression is detrimental to the host in the absence of caspase-1 during *S. typhimurium* infection. The mechanism behind these results is still largely unknown. However, we made two key observations. First, caspase-1 expression is associated with efficient neutrophil clearance of *S. typhimurium* (55). Second, *Casp1*^*−/−*^ mice contain large mats of extracellular bacteria in their liver that are not observed in wildtype, *Casp11*^*−/−*^, or *Casp1*^*−/−*^*Casp11*^*−/−*^ mice (55). These results indicate that caspase-11 expression leads to host cell lysis and release of *S. typhimurium*. Extracellular *S. typhimurium* are subsequently recognized and efficiently cleared by caspase-1-regulated neutrophils. The mechanism of how caspase-1-mediated function is affecting the ability of neutrophils to phagocytose bacteria is unknown and an area worth pursuing.

Previous studies demonstrate an essential role for caspase-11 in response to cytosolic LPS (59). This result led to the evaluation of cytosolic gram-negative bacterial pathogens and caspase-11. Infections with the naturally cytosolic gram-negative bacterial pathogens, *Burkholderia thailandensis* and *Burkholderia pseudomallei*, leads to caspase-11-dependent cell death in BMDMs (81). In addition, caspase-11 was found to protect against a lethal infection by *B. thailandensis* (81). To further expand upon these findings, the authors took advantage of two naturally occurring vacuolar pathogens, *S. typhimurium* and *L. pneumophila*, and evaluated mutants (Δ*sifA* and Δ*sdhA*, respectively) that were previously demonstrated to rupture the vacuole and release these Gram-negative pathogens into the cytosol of host cells (82,83). Interestingly, *S. typhimurium* Δ*sifA* and *L. pneumophila* Δ*flaA*Δ*sdhA* (a strain that also lacks flagellin and thus fails to trigger the NLRC4 inflammasome) increased cell death in BMDMs that is dependent on caspase-11 (81). Further *in vivo* studies demonstrated that caspase-11 expression promotes bacterial clearance of *S. typhimurium* Δ*sifA* but not wildtype *S. typhimurium* (81). These results provide an explanation for why *S. typhimurium* Δ*sifA* is attenuated *in vivo* (82). Moreover, these results are consistent with caspase-11 either directly or indirectly sensing LPS.

## Cell type-specific inflammasome activation and regulation

### Inflammasome activity in epithelial defenses

The primary effectors of inflammasome-mediated control of bacterial infections are thought to be immune cells. However, two recent studies have demonstrated that intestinal epithelial cells (iECs) are also an important source of inflammasome-mediated defense against *S. typhimurium* (84,85). iECs lie at the interface between the intestinal lumen and immune cells and provide a physical barrier to invading bacterial pathogens. *S. typhimurium* occupy two distinct populations within iECs. One population resides within the membrane-bound *Salmonella* containing vacuole (SCV), while a small subset can escape the SCV and replicate in the cytosol (86). Epithelial cells containing cytosolic *S. typhimurium* lyse, release IL-18, and are extruded from the monolayer (86). Because of these known inflammasome outputs, the role of epithelial cell-based inflammasome-mediated defense against *S. typhimurium* was examined (85). Oral infection of streptomycin-pretreated mice results in rapid gut colonization and proliferation followed by a reduction in bacterial loads 18 h postinfection (85). This bacterial decline correlated with the expulsion of epithelial cells containing *S. typhimurium*, suggesting iEC expulsion may restrict *S. typhimurium* replication (85). Indeed, compromising epithelial cell expulsion results in higher bacterial burdens (85). The epithelial-specific bacterial restriction is dependent on the inflammasome components NAIP1-6 and NLRC4, but not on the downstream cytokines IL-18 and IL-1B (85). In addition, a second report demonstrated that caspase-11 also plays an important role in epithelial defense to *S. typhimurium* by inducing epithelial cell shedding (84). The bacterial burdens of *Casp11*^*−/−*^ mice infected with *S. typhimurium* are elevated in the intestine but not in systemic sites, consistent with previous reports (84). *Casp11*^*−/−*^ mouse intestines are littered with epithelial cells containing *S. typhimurium* microcolonies, a phenotype rarely observed in wildtype mice (84). Consistently, caspase-4 in human iECs is important for restricting cytosolic bacterial replication of *S. typhimurium* (84). This response is dependent on cell shedding but independent of IL-18 (84). These results begin to characterize the factors involved in accelerated gut epithelial cell shedding during *S. typhimurium* infection and are potentially a common mechanism to limit other enteric pathogens.

One notable difference in inflammasome activity in iECs is that caspase-4, but not caspase-1 or caspase-5, is required for IL-18 secretion and processing in response to *S. typhimurium* and EPEC infection (84). Consistently, cecal contents of *Casp11*^*−/−*^ mice infected with *S. typhimurium* are reduced for IL-18 levels (84). These results were unexpected because activation of caspase-11 in murine macrophages results in cell death, but only plays a minor role in cytokine processing (55). Nevertheless, similar to BMDMs, cytosolic LPS activates caspase-4 in human iECs (84). These results highlight variations between inflammasome outputs in different cell types despite the same stimulus.

### Inflammasome activity in dendritic cell upon infection

Macrophages and dendritic cells (DCs) are phagocytic cells that are critical players in the immediate response to infection. Both cell types are efficient at taking up foreign products, processing them, and mounting an appropriate inflammatory response. Dendritic cells bridge the immediate, preprogrammed arm of the immune system with the highly pathogen-specific adaptive immune response. The overwhelming majority of *in vitro* inflammasome research has been done in murine macrophages, but there is good reason to suspect that macrophages and DCs differ in their expression of inflammasome components and/or in their responses to bacterial PAMPs. For example, *L. pneumophila* can replicate in macrophages, but its replication is restricted in DCs by induction of apoptosis (87,88).

To further compare and contrast macrophages and DCs, we investigated inflammasome activation in BMDCs in response to *F. novicida* infection (89). Similar to BMDMs, *F. novicida* is phagocytosed and initially resides within the FCV. By 4 h postinfection, the majority of bacteria escape the FCV and begin rapidly replicating within the host cytosol. In both BMDMs and BMDCs, escape from the FCV and bacterial replication requires the FPI (89). Interestingly, *F. novicida* infection of BMDCs initiates a rapid inflammasome-mediated cell death, characterized by high levels of LDH release, IL-1β secretion and caspase-1 processing (89). Like BMDMs, BMDCs derived from ASC-, AIM2-, or caspase-1-deficient mice are defective for cell death, IL-1β secretion and caspase-1 processing in response to infection with wildtype *F. novicida* (89). In addition, BMDCs infected with *F. novicida* prelabeled with Hoechst DNA stain contain multiple DNA-AIM2 specks, likely reflecting AIM2 interacting with DNA released from lysed bacterial cells (89). Despite the presence of many AIM2-DNA foci within a single BMDC, only one AIM2-DNA-ASC complex forms, similar to our previous reports in macrophages (35). While the core inflammasome components necessary to respond to *F. novicida* infection appear to be similar between macrophages and DCs, we did find a difference in the requirement for type I IFN signaling and inflammasome activation. BMDMs deficient in the type I IFN receptor 1 (*IFNAR1*) do not mount an AIM2 inflammasome response to *F. novicida* infection (30,35,90). In contrast, *IFNAR1*-deficient BMDCs are only partially inhibited for caspase-1-mediated cell death and IL-1β secretion, demonstrating a differential requirement for type I IFN signaling and inflammasome activation (89). Future studies are needed to address the precise mechanisms of communication between inflammasome activity and type I IFN signaling in different cell types.

## Regulation of inflammasomes

### NF-κB activation by LPS

Inflammasome activation and pro-IL-1β levels are highly influenced by pro-inflammatory signaling pathways such as those triggered by the TLR pathway. Many TLRs signal through the MyD88 adapter to activate the NF-κB transcription factor, resulting in elevated pro-IL-1β levels and *NLRP3* expression (4). During a typical bacterial infection, several TLRs can be activated by a variety of bacterial ligands to result in NF-κB-dependent signaling. However, LPS stimulation is commonly used as a prototypical bacterial agonist to ‘prime’ inflammasome components for activation by a second signal. Upon inflammasome activation, caspase-1 cleaves pro-IL-1β and pro-IL-18 to their active forms IL-1β and IL-18, respectively, and releases the cytokines into the extracellular milieu through an uncharacterized mechanism (4). Consequently, secretion of the pro-inflammatory cytokines is controlled by a two-step mechanism, transcriptional priming followed by posttranscriptional processing.

### Type I IFN signaling

**Fig. 3:**
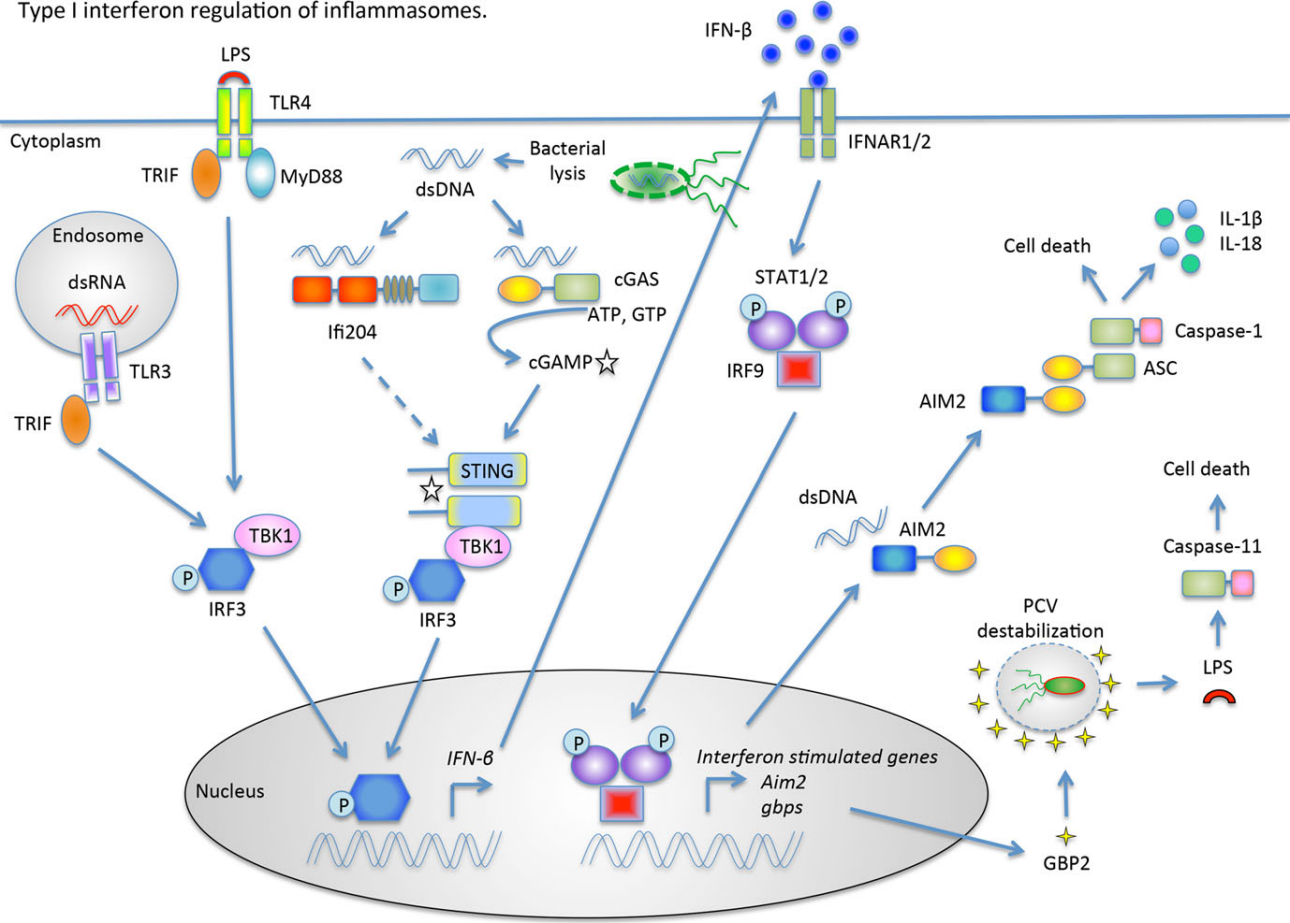
Type I IFN regulation of inflammasomes during bacterial infection Type I IFN signaling can be induced through TLR4, located on the plasma membrane, TLR3, located within endosomes, or through DNA sensors, located in the cytosol. During a *F. novicida* infection, a subset of bacteria lyse within the cytosol and activate the DNA sensors, Ifi204 and cGAS, which trigger a STING- and IRF-3-dependent production of type I interferons. Autocrine and paracrine signaling through the type I interferon receptor (IFNAR) leads to STAT1/2 activation and induction of interferon stimulated genes, among them *AIM2* and *Gbps*. AIM2 recognizes cytosolic DNA and interacts with ASC to activate caspse-1. GBP2 associates with the pathogen containing vacuole (PCV) promoting the lysis of the PCV through an unknown mechanism. Upon PCV degradation, bacteria are released into the cytosol and can activate caspase-11 by directly binding bacterial LPS.

Host cells respond to the presence of invading pathogens with the production of type I interferons (IFNs) (28). Type I IFNs (e.g. IFNα and IFNβ) are a family of secreted cytokines that act early in the innate immune response. Although type I IFNs are best known for their ability to elicit an effective antiviral response, they have recently been shown to play an important role in the host response to bacterial infections (28,91). Importantly, specific inflammasomes can be influenced by the production of type I IFNs (*Fig.* 3).

Two general pathways for type I IFN induction in immune cells have been described (28). First, type I IFNs are produced in response to dsRNA, ssRNA, and LPS, which activate the transmembrane receptors TLR3, TLR7/8, and TLR4, respectively (92). They signal through the TRIF adapter to activate the transcription factor IRF-3 (92). The second pathway is TLR-independent and relies on the activation of cytosolic receptors in response to cytosolic PAMPs, such as nucleic acids and bacterial cell wall components. In many instances, cytosolic receptors signal through the endoplasmic reticulum membrane protein stimulator of IFN genes (STING, also known as MITA, MPSY, ERIS, and TMEM173). STING activation leads to the recruitment of the kinase TBK1, which then activates the IRF-3 transcription factor, leading to the induction of *IFN-β1* (93,94). Once produced, type I IFNs signal in an autocrine and paracrine manner through a common receptor IIFNAR, which is composed of a heterodimer of IFNAR1 and IFNAR2. Upon ligand-induced receptor dimerization, IFNAR stimulates the Janus kinase 1 (JAK1) signal transduction pathway leading to the transcriptional response of IFN-stimulated genes (ISGs) containing an IFN-stimulated response element (ISRE) in their promoter region. Nearly 3000 ISGs have been identified thus far, and many participate in mounting a strategic host defense (95).

### Type I IFNs activate the AIM2 inflammasome

Many intracellular bacterial pathogens elicit a STING-dependent type I IFN host response, including *F. novicida*, *L. monocytogenes, M. tuberculosis*, and C*. trachomatis* (96–99). Our previous experiments with *F. novicida* identified a link between type I IFN signaling and inflammasome activation (100). Macrophages infected with cytosolic *F. novicida* elicit a robust type I IFN response that is required for caspase-1 processing, IL-1β secretion, and cell death in BMDMs (100). Macrophages deficient for type I IFN signaling (*STING*^*−/−*^, *IRF-3*^*−/−*^ and *IFNAR*^*−/−*^) do not process caspase-1, secrete IL-1β or die in response *F. novicida* infection (30,35,100). Type I IFN signaling is also necessary for inflammasome activation by the cytosolic pathogen *L. monocytogenes*, but not the vacuolar pathogen *S. typhimurium* (100). This discrepancy in inflammasome activation by different bacterial species is attributed to the AIM2 inflammasome, which is activated by *F. novicida* and *L. monocytogenes* but not *S. typhimurium* (30,35,49). *AIM2* is an interferon-inducible gene whose gene product serves as a dsDNA inflammasome sensor (29,36). IFN-β priming of macrophages enhances AIM2-dependent cell death in response to *F. novicida* infection (35). Consistent with type I IFN signaling functioning upstream of inflammasome activation, priming *STING*^*−/−*^ or *IRF-3*^*−/−*^ macrophages with IFN-β restores AIM2 inflammasome activation upon *F. novicida* infection (35). These findings demonstrate the importance of type I IFN signaling in *F. novicida*-mediated AIM2 inflammasome activation.

While type I IFN signaling is appreciated as an important host-pathogen signaling pathway, little is known about the bacterial ligands and corresponding host receptors that trigger type I IFN production. We were interested in identifying both the bacterial ligand(s) and host factor(s) involved in the type I IFN response during a *F. novicida* infection. Unlike typical gram-negative bacteria, *F. novicida* has a modified LPS structure that fails to interact with TLR4 (101). Initial experiments determined that *F. novicida* infection primarily stimulates the TLR-independent type I IFN pathway as *Myd88*^*−/−*^*TRIF*^*−/−*^ double-knockout macrophages are only slightly reduced for *INF-β1* transcription in response to *F. novicida* infection (90). Rather, the type I IFN response to *F. novicida* is almost solely STING-dependent and is necessary to activate the AIM2 inflammasome (35,90,102).

STING-dependent signaling is essential for the type I IFN response to cytosolic DNA (103). DNA can enter the cytosol either through DNA transfection or from bacterial species that access the cytosol likely through inadvertent bacterial lysis (see dsDNA) (103). We and others (35,48,102) showed that *F. novicida* releases dsDNA into the macrophage cytosol during infection. We hypothesized that a DNA sensor is necessary to facilitate the STING-dependent type I IFN response. To test this notion, we knocked down the expression of known DNA sensors that are important for triggering a type I IFN response (104–108). They include RNA polymerase III, DNA-dependent activator of IFN-regulatory factors (DAI), Lrrfip1, Ifi204 (human: IFI16), Mre11, DNA-dependent protein kinase (DNA-PK), cyclic GMP-AMP synthase (cGAS), and Ddx41 (104,106–112). We found that upon *F. novicida* infection, siRNA knockdown of *cGAS* and *Ifi204* resulted in reduced type I IFN production, while siRNA knockdown of *Lrrfip1*, *RIG-I,* and *Ddx41* did not influence the type I IFN response (authors’ unpublished data). Consistent with the fact that AIM2 inflammasome activity is regulated by type I IFNs, *cGAS*^*−/−*^ BMDMs infected with *F. novicida* released less IL-1β, had less processed caspase-1 and lower levels of cell death compared to wildtype BMDMs (authors’ unpublished data).

Both cGAS and Ifi204 are proposed cytosolic DNA sensors. Upon DNA recognition, cGAS activates its nucleotidyltransferase activity to produce the host-derived second messenger cyclic GMP-AMP (cGAMP) (93,108). cGAMP and the closely related bacterial-derived cyclic-dinucleotides, c-di-GMP, and c-di-AMP, directly activate STING to elicit a type I IFN response (93,97,108). In contrast, the mechanism of Ifi204-dependent activation of STING is less clear. The human orthologue of Ifi204, IFI16, interacts with STING upon DNA stimulation (107). In addition, Ifi204 and STING co-localize when co-transfected in HeLa cells (113). Since cGAS and Ifi204 have both been implicated in the sensing of cytosolic dsDNA, we hypothesized that the type I IFN-stimulating PAMP during *F. novicida* infection was DNA (109). To test this, we treated *F. novicida* lysates with DNase or RNase and transfected the treated lysates into murine macrophages. *F. novicida* lysates stimulated a robust type I IFN response that was abolished when the lysates were treated with DNase I, but not with RNase A, suggesting DNA is the primary type I IFN stimulus (authors’ unpublished data). Therefore, *F. novicida* DNA in the cytosol appears to activate two host responses. First, *F. novicida* DNA activated the STING-dependent type I IFN response through cGAS and Ifi204 (authors’ unpublished data). Second, *F. novicida* DNA activates the AIM2 inflammasome (35). These studies underscore the importance of DNA recognition during a bacterial infection.

### Type I IFNs stimulate GBPs to activate caspase-11

Type I IFN signaling is important for activating caspase-11 (55,56). Macrophages deficient for the TRIF signaling adapter fail to induce caspase-11-dependent cell death in response to Gram-negative bacteria, suggesting type I IFN production to be important for caspase-11 activation (55,56). However, type I IFN production is not required for pro-caspase-11 protein expression or caspase-11-dependent cell death upon LPS transfection (59,114). These results demonstrate that type I IFN production is only important in the context of a bacterial infection, suggesting ISG(s) are important factors in facilitating access of the activating ligand (LPS) to caspase-11. Among the most highly induced proteins during *S. typhimurium* infection is a group of IFN-induced GTPases (114). IFN-induced GTPases consists of four subfamilies of GTPases including immunity-related GTPases (IRGs), guanylate-binding proteins (GBPs), myxoma (MX)-resistant proteins, and the very large inducible GTPases (VLIGs/GVINs). These proteins function to restrict pathogen replication, induce host cell death, and have been associated with inflammasome activation (95,115,116). Indeed, macrophages deficient for *Gbps* on chromosome 3 (*Gbp1, 2, 3, 5,* and *7*) are reduced for caspase-11-dependent cell death in response to the gram-negative vacuolar pathogens *S. typhimurium* and *L. pneumophila* (114,117). Individual siRNA knockdowns identified Gbp2 as the primary GTPase involved in caspase-11-mediated cytotoxicity in response to *S. typhimurium* infection (114). These GBPs are important for controlling *S. typhimurium* and *L. pneumophila* replication (114,117).

Physical association of GBPs with pathogen-containing vacuoles is believed to be necessary in restricting bacterial replication by GBPs was hypothesized to require GBPs physically associating with pathogen-containing vacuoles (95). Indeed, GBP2 associates with the SCV and aids in its destabilization through an unclear mechanism, leading to higher numbers of cytosolic *S. typhimurium* (114). The cytosolic *S. typhimurium* population subsequently activates caspase-11 through LPS detection (114). These reports highlight a link between type I IFN production, GBPs, and caspase-11 inflammasome activation by gram-negative bacteria. GBP5 was also reported to be an important activator of the NLRP3 inflammasome in response to *L. monocytogenes* and *S. typhimurium* infections when induced with IFN-γ (116), but there are no differences in NLRP3 activation in the absence of prestimulation (114). GBPs are also important mediators of host defense against non-gram-negative pathogens, including *Toxoplasma gondii*, *L. monocytogenes*, and *M. bovis* BCG (115,118). While GBPs play a critical role in host defense, an undisputed mechanism of how GBPs contribute to pathogen defense has yet to emerge. Future studies examining the role of GBPs in a wide range of microbial infections may identify additional type I IFN links to inflammasomes.

## *Salmonella* manipulates the host environment to favor replication

Host-adapted *Salmonella* serovars are able to persist within hosts for long periods of time (119). We have found in a mouse model that *S. typhimurium* persists within mouse macrophages in systemic tissues, suggesting that macrophages are an important niche for persistence (120). Macrophages exist in a spectrum of activation states. Traditionally, macrophage activation states are divided into two major categories designated M1 and M2. Classically activated (M1) macrophages are highly inflammatory, while alternatively activated (M2) macrophages are less inflammatory and are involved in tissue repair. We demonstrated that *S. typhimurium* preferentially associates with M2 macrophages at later stages of disease and that there is very little host cell death (121). Although the mechanisms that promote *Salmonella* persistence are not known, one possible contributing factor is that M2 macrophages, which are considerably less inflammatory than M1 macrophages, are less prone to inflammasome activation in response to *S. typhimurium*. Reduced inflammasome activation could lead to enhanced cell survival by providing a replicative niche for *S. typhimurium*. In support of this notion, one study found that elimination of the NLRP3 inflammasome in 9-month-old C57Bl/6 obese mice increases the number of M2 macrophages but does not affect the number of M1 macrophages (122). In addition, obese ZDF rats have higher caspase-1 activity and increased mRNA expression of *Nlrp3*, *Il1β*, and *Il18* (123). Interestingly, treatment with the CB_1_R inverse agonist JD5037, which reduces obesity, causes a shift from M1-to-M2 macrophages and correspondingly reduced mRNA expression levels of *Nlrp3*, *Il1β* and *Il18* (123). Collectively, these results suggest that the M2-like macrophages express fewer inflammasome components, which may be an additional factor that drives intracellular *S. typhimurium* replication and persistence.

## Inflammasome activation consequences

Caspase-1 activation leads to two outcomes: pyroptosis and cytokine secretion. In this section, we discuss effector functions attributed uniquely to each of the two outcomes. Both pyroptosis and cytokine secretion can aid the host in defending against bacterial pathogens.

### Pyroptosis

Pyroptosis, or caspase-1-dependent programmed cell death, results in rapid swelling and lysis of the affected cell (7). The inflammatory nature of pyroptotic cell death is one characteristic that differentiates it from apoptosis (7). Pyroptosis helps to restrict the growth of intracellular pathogens. This type of cell death is observed upon infection by a variety of intracellular bacteria including *Francisella*, *Legionella*, and *Yersinia* (34,70,124). Pyroptosis effectively short-circuits the pathogen replication cycle and releases the intracellular pathogens prematurely from the macrophage. Released bacteria are subsequently exposed to additional host defense systems, including phagocytosis by neutrophils. Intracellular replication is required for *F. novicida* pathogenicity (45). Thus, *Casp1*^*−/−*^ and *ASC*^*−/−*^ BMDMs, which do not undergo pyroptosis, support higher bacterial burdens of *F. novicida* compared to wildtype BMDMs (34). To determine if pyroptosis or cytokine secretion was primarily responsible for the increased susceptibility of *Casp1*^*−/−*^ mice, we treated wildtype mice with both IL-1β and IL-18 neutralizing antibodies. Wildtype mice treated with IL-1β and IL-18 neutralizing antibodies are more susceptible to *F. novicida* infection compared to mice treated with control antibodies; however, they are not as susceptible as caspase-1-deficient mice (34). In addition, *Casp1*^*−/−*^ mice are protected from endotoxin-induced mortality, whereas *IL-1β*^*−/−*^*IL-18*^*−/−*^ double knockout mice are not (125). These studies provide some evidence that inflammasome activation has effects *in vivo* that cannot be solely accounted for by IL-1β and IL-18 cytokines. Upon further analysis, susceptibility to endotoxin-induced mortality is dependent on caspase-11 (59,79). Administration of neutralizing antibodies against the alarmin HMGB1 enhances survival in the endotoxin-induced septic shock model (126). Thus, it suggests that endotoxin-induced mortality is predominantly due to caspase-11-dependent release of HMGB1 and potentially other sepsis mediators. Interestingly, treatment with neutralizing antibodies against HMGB1 also protected BALB/c mice against infection by the most virulent *Francisella* subsp. *tularensis* (127). Since *Francisella* does not activate caspase-11 (128), the role of caspase-1 and HMGB1 release *in vivo* remains to be determined.

An additional benefit to inflammasome-induced pyroptosis was recently identified to be the release of polymerized ASC into the extracellular space (129). Upon ligand recognition by an inflammasome sensor, ASC is rapidly polymerized, forming an ASC speck that is readily visualized by microscopy (130). When cells die via pyroptosis, their intracellular contents are released into the extracellular space. Interestingly, released ASC specks remain active for an extended period of time where they continue to promote the maturation of IL-1β (129). Furthermore, naïve macrophages that phagocytose extracellular ASC specks become activated and promote soluble ASC polymerization, lysosomal damage and IL-1β secretion in recipient cells (129). The ability of cells to transfer ASC aggregates by pyroptosis represents a novel mechanism for cell-to-cell communication. However, the relevance of extracellular ASC during bacterial infection is not yet known.

### Cytokine production

Cytokines are key mediators of the immune system. The cytokines IL-1β and IL-18 are produced in response to inflammasome activation and induce a powerful pro-inflammatory response with pleiotropic effects. IL-1β and IL-18 have amino terminal pro-domains that require cleavage by caspase-1 to generate the bioactive form. Upon cleavage, IL-1β and IL-18 are released from the cell through an unidentified mechanism and signal through the receptors IL-1 receptor (IL-1R) and IL-18R, respectively. The IL-1 receptors trigger a MyD88- and TRAF6-dependent signaling pathway that ultimately activates signal transduction pathways such as NF-κB and MAPK resulting in the release of additional pro-inflammatory cytokines, such as TNFα and IL-6 (131). In addition, IL-18 stimulates IFNγ production from T and NK cells promoting the antimicrobial activity of macrophages by inducing nitric oxide production (132). IL-1β can also provide protection against infections by activating several host responses including neutrophil recruitment and stimulating the Th17 response (132).

Caspase-1-deficient mice are more susceptible to many bacterial pathogens. In particular, *Casp1*^*−/−*^ mice infected by *Shigella*, *Francisella*, *Salmonella*, *Listeria*, and *Legionella* have higher bacterial loads compared to wildtype mice (133). One important question is whether the caspase-1 substrates IL-1β and IL-18 specifically contribute to the antimicrobial phenotype apparent in caspase-1-deficient mice. Almost invariably, both IL-1β and IL-18 are found to be protective (132). In the case of *S. typhimurium*, caspase-1 provides protection from both oral and IP infection (134). In addition, IL-1β and IL-18 contribute to the control of *S. typhimurium* as both *IL-1β*^*−/−*^ and *IL-18*^*−/−*^ mice are more susceptible to oral *S. typhimurium* infection compared to wildtype mice (134). Interestingly, only IL-18 appears to contribute to resistance to systemic *S. typhimurium* (134), suggesting that IL-1β is important for protection in the gut but not systemic sites, while IL-18 appears to be the crucial factor in immune protection against *S. typhimurium* regardless of route of inoculation. Similarly, IL-18 but not IL-1β, are important in protection against *Shigella* and *Listeria* infections (69,71,135). These results provide evidence that IL-1β and IL-18 directly contribute to bacterial resistance.

## Microbiota and inflammasomes

Extensive work has characterized inflammasomes as mediators of inflammation in response to pathogens. However, it is becoming more evident that certain inflammasomes are critical for maintaining intestinal homeostasis. Specifically, both NLRP6 and NLRP3 deficiencies are linked to exacerbation of chemical-induced colitis (136,137). In particular, NLRP6-deficient mice have altered fecal microbiota characterized by an expanded representation of the bacterial phylum *Bacteroidetes* and TM7 but decreased representation of members in the *Lactobacillus* genus compared to wildtype mice (137). The altered fecal microbiota in *Nlrp6*^*−/−*^ mice resembles that of *ASC*^*−/−*^ and *Casp1*^*−/−*^ mice, suggesting the involvement of the inflammasome in this phenotype (137). Interestingly, both the *Bacteroidetes* and TM7 phyla are associated with IBD in humans (138). Thus, it was not surprising that the NLRP6 inflammasome was found to play a critical role in preventing chemical-induced colitis [dextran sodium sulfate (DSS) treatment] as *Nlrp6*^*−/−*^ and *ASC*^*−/−*^ mice display increased exacerbation of disease characterized by increased spontaneous intestinal hyperplasia, elevated inflammatory cell recruitment, increased weight loss and reduced survival (137). During chemical-induced colitis, gut epithelial cells are disrupted and allow the dissemination of commensal bacteria into the underlying tissue promoting extensive inflammation. A hallmark of inflammasome activation is the secretion of the pro-inflammatory cytokines IL-1β and IL-18. Somewhat surprisingly, only *IL-18*^*−/−*^ mice but not *IL-1β*^*−/−*^ or *IL-1R*^*−/−*^ mice are more susceptible to chemical-induced colitis, suggesting that IL-18 is a major contributor to microbiota homeostasis (137). Consistent with IL-18 playing an important role in resisting chemical-induced colitis, *IL-18*^*−/−*^ mice have increased expansion of the *Bacteroidetes* phylum, and *Nlrp6*^*−/−*^ mice have reduced basal serum levels of IL-18 (137). However, *IL-18*^*−/−*^ mice contain differences in microbiota composition to *Nlrp6*^*−/−*^ mice, suggesting that other inflammasome factors are also involved in shaping the intestinal microbiota (137).

Enhanced susceptibility to chemical-induced colitis has also been demonstrated for the NLRP3 inflammasome. Upon chemical-induced colitis treatment, *Nlrp3*^*−/−*^ mice lose epithelial cell integrity resulting in the dissemination of commensal bacteria, massive leukocyte infiltration, increased cytokine production, and ultimately higher mortality rates compared to wildtype mice (136). Like the NLRP6 inflammasome, the increased susceptibility of *Nlrp3*^*−/−*^ mice to chemical-induced colitis is attributed to lower levels of IL-18 (136). In addition, previous studies demonstrated a link between decreased *NLRP3* expression and increased susceptibility to Crohn's disease in humans (139). Taken together, the NLRP3 inflammasome is important for the preservation of intestinal homeostasis and protection against excessive inflammation in the gut.

NLRP6 and NLRP3 inflammasomes are both important for resisting DSS-induced colitis (136,137). However, these inflammasomes play different roles when infected with intracellular pathogens. Experiments from our laboratory determined NLRP3 is important for resisting *S. typhimurium* infection as *Nlrp3*^*−/−*^*Nlrc4*^*−/−*^ double knockout mice contain higher bacterial burdens in systemic sites compared to wildtype mice (74). These results suggest that the NLRP3 inflammasome helps to control *S. typhimurium* infection. In contrast, the NLRP6 inflammasome confers sensitivity to the bacterial pathogens, *S. typhimurium, L. monocytogenes*, and *E. coli* (140). *Nlrp6*^*−/−*^ mice carry significantly lower bacterial burdens in both the spleen and liver when infected by IP injection with intracellular pathogens, *S. typhimurium* and *L. monocytogenes*, and extracellular pathogens, *E. coli* (140). NLRP6 specifically inhibits TLR2- and TLR4-dependent activation of NF-κB and MAPK pathways (140). Consequently, *Nlrp6*^*−/−*^ BMDMs and *Nlrp6*^*−/−*^ mice produce elevated levels of TNF-α, IL-6, and KC in response to bacterial infection or TLR2 or TLR4 agonists (140). In addition, higher numbers of circulating monocytes and neutrophils were present in the peripheral blood of *Nlrp6*^*−/−*^ mice infected by IP with *L. monocytogenes*, suggesting a role for NLRP6 in dampening the immune response to bacterial pathogens (140).

In contrast to IP infections, oral infections with the enteric pathogen *Citrobacter rodentium* resulted in worse pathology and increased bacterial burdens in the gut tissues in *Nlrp6*^*−/−*^ mice (141). This altered susceptibility is not due to the cellular response as IL-1β and IL-18 expressions from the distal colon are similar in wildtype and *Nlrp6*^*−/−*^ mice (141). However, the NLRP6 inflammasome is important for regulating mucus secretion from colonic goblet cells (141). The mechanism proposed was that a NLRP6 deficiency reduces mucus production by inhibiting the autophagic pathway necessary for proper exocytosis of mucus granules (141). The detrimental role of NLRP6 in the context of a bacterial IP infection appears in sharp contrast to its protective function in preventing colitis in the gut and protecting against gut infections (137,140). Further studies will be important to clarify these discrepancies.

To date, the ligand that activates the NLRP6 inflammasome has not been identified. Initial over-expression studies demonstrate that IL-1β is secreted from COS-7L cells when NLRP6 (PYPAF5), ASC and pro-caspase-1 are co-expressed (142). These studies suggest that NLRP6 can form a functional inflammasome typical of other NLR proteins. However, NLRP6 is dispensable for inflammasome activation in response to *L. monocytogenes* and *S. typhimurium* infection as *Nlrp6*^*−/−*^ BMDMs process caspase-1 and secrete IL-1β similar to wildtype macrophages (140).

## Concluding remarks

The innate immune system is crucial for an effective host defense against invading pathogens. Understanding how pathogens are recognized and how innate immune systems respond is the bread and butter of bacterial pathogenesis. The identification of inflammasomes was a critical turning point in understanding host-pathogen interactions. Inflammasomes are molecular platforms that are important in mounting an effective host response against bacteria, viruses, fungi, and parasites. In addition, dysregulation of inflammasomes have been linked to human inflammatory diseases, such as type 2 diabetes, atherosclerosis, vitiligo, celiac disease, and gout (143). Although we know that the proper regulation of inflammasome activation is central for human health, there are many unanswered questions. For example, only a handful of inflammasome sensors have been characterized, and the identification of their cognate ligands will no doubt have an important impact on the control of infectious diseases. In addition, characterizing the factors involved in inflammasome regulation and signaling will lead to the identification of novel targets for therapeutic intervention in infectious, autoinflammatory, and autoimmune diseases.
